# Tumor-Associated Microbiota in Proximal and Distal Colorectal Cancer and Their Relationships With Clinical Outcomes

**DOI:** 10.3389/fmicb.2021.727937

**Published:** 2021-09-28

**Authors:** Min Jin, Fumei Shang, Jingjing Wu, Qilin Fan, Chen Chen, Jun Fan, Li Liu, Xiu Nie, Tao Zhang, Kailin Cai, Shuji Ogino, Hongli Liu

**Affiliations:** ^1^Cancer Center, Union Hospital, Tongji Medical College, Huazhong University of Science and Technology, Wuhan, China; ^2^Department of Medical Oncology, Nanyang Central Hospital, Nanyang, China; ^3^Department of Pathology, Union Hospital, Tongji Medical College, Huazhong University of Science and Technology, Wuhan, China; ^4^Department of Epidemiology and Biostatistics, The Ministry of Education Key Lab of Environment and Health, School of Public Health, Huazhong University of Science and Technology, Wuhan, China; ^5^Department of Gastrointestinal Surgery, Union Hospital, Tongji Medical College, Huazhong University of Science and Technology, Wuhan, China; ^6^Program in Molecular Pathological Epidemiology, Department of Pathology, Brigham and Women’s Hospital and Harvard Medical School, Boston, MA, United States; ^7^Department of Epidemiology, Harvard T.H. Chan School of Public Health, Boston, MA, United States; ^8^Broad Institute of MIT and Harvard, Cambridge, MA, United States; ^9^Cancer Immunology and Cancer Epidemiology Programs, Dana–Farber Harvard Cancer Center, Boston, MA, United States

**Keywords:** distal colorectal cancer, proximal colon cancer, microbiota, 16S rRNA, Fusobacteria, clinical outcomes

## Abstract

The proximal and distal subsites of colorectal cancer (CRC) have distinct differences in their embryonic origin, epidemiology, and prognosis. Therefore, they are not considered as the same disease. However, the possible difference in microbial characterization of the two subsites of CRC is still unclear. In this study, we explored tumor microbiota diversity and composition difference in patients with proximal (*N* = 187) and distal CRCs (*N* = 142). This was carried out on cancer tissues and adjacent tissues using bacterial 16S rRNA sequencing. The Kaplan–Meier method was used to analyze the correlation between differential flora and overall survival rate of the patients. It was found that there were significant differences in tumor microbial characteristics between the proximal and distal CRC tissues. The microbiota communities were distinctly richer in the proximal colon tumor tissues than in the distal CRC tissues. Microbial diversity and structure were relatively constant in the paracancerous normal tissues of the proximal and distal colorectum. Generally, microbial communities of CRC tumor tissues were composed of Proteobacteria, Firmicutes, Actinobacteria, and Bacteroidetes. Alpha diversity in the proximal and distal CRC tumor tissues was closely related to specific microflora. The abundance of Fusobacteria was associated with age of patient, tumor diameter, and tumor microsatellite instability (MSI) status of the patients. Moreover, Fusobacteria enrichment was associated with poor prognosis especially in patients with proximal colon cancers, but not in patients with distal CRC. In conclusion, proximal and distal subsites of the CRC present distinct microbiota diversity and community structures. The differences indicate that there are different risk factors across anatomical subsites of CRC, which may provide a new strategy for precise prevention and treatment of CRC in the future.

## Introduction

Despite improvements in screening, prevention, and treatment strategies over the past decade, colorectal cancer (CRC) is the third leading malignant cancer and the fourth cause of cancer-related deaths. It is estimated that 1,849,518 new cases of CRC and 880,792 CRC-related deaths occur per year (Bray et al., 2018). This is despite notable improvements in the disease screening, prevention, and treatment strategies over the past decade. CRC originating proximally (right) or distally (left) exhibits differences in embryonic origin, age-specific and sex-specific morbidity, clinical manifestation, pathological characteristics, and development (Petrelli et al., 2017; Cannon and Buechler, 2019). At the molecular level, *BRAF* mutation, microsatellite instability (MSI), the CpG island methylator phenotype (CIMP)-high, or the consensus molecular subtype (CMS) CMS1 is more likely to occur in proximal CRC (Missiaglia et al., 2014; Guinney et al., 2015; Lee et al., 2017), while chromosome instability, *TP53* or *APC* mutation, or CMS2 is more likely to occur in distal CRC (Missiaglia et al., 2014; Guinney et al., 2015; Loree et al., 2018). Moreover, proximal CRC commonly has a poorer prognosis than distal CRC (Petrelli et al., 2017; Cannon and Buechler, 2019). These differences between anatomical segments reflect a complex interaction between different environmental exposures of colorectal epithelial cells to carcinogenic or protective factors and the different inherent biological features affecting the risk of anatomical subsite carcinogenesis.

Overwhelming evidence indicates that gut microbiota play a critical role in the development of colorectal malignancies (Brennan and Garrett, 2016; Thomas et al., 2019). It has been found that more than 100 trillion (10^14^) microorganisms inhabit the host gastrointestinal tract and maintain tissue homeostasis (Eckburg et al., 2005; Ley et al., 2006). In addition to influencing gut nutrition and metabolism, the intestinal microbiota can play both beneficial and harmful roles *via* either their metabolites or interaction with their host intestinal epithelial cells (Bullman et al., 2017). Disruption of intestinal homeostasis can cause a series of intestinal diseases, including inflammatory bowel diseases (Larabi et al., 2019), colonic adenoma (Thomas et al., 2019), and neoplasia. The mechanisms of bacteria contributing to colorectal tumors are complex and may involve chronic inflammation, direct and indirect effects on host cells, and interactions with tumor microenvironments.

Recently, several studies have identified a series of special bacterial species involved in the colorectal carcinogenesis, and some bacteria derived the carcinogenesis *via* a dubbed “driver–passenger” model (Tjalsma et al., 2012). *Streptococcus gallolyticus* was the first reported bacterium associated with CRC presenting approximately 18–62% of CRC and less than 5% in the normal colon. Abdulamir et al. (2009) reported that NF-κB and IL-8 act as key mediators for the *S. gallolyticus*-associated carcinogenesis of adenoma to carcinoma. *Helicobacter pylori* is closely associated with gastric cancer (Sokic-Milutinovic et al., 2015) and has recently been listed as a gastrointestinal carcinogen by the International Agency for Research on Cancer (IARC). Zumkeller et al. (2006) showed a 1.4-fold increased risk of CRC in patients infected with *H. pylori*. Additionally, *Fusobacterium nucleatum* is an anaerobic Gram-negative pathogen, and its enrichment in CRC tissues is associated with shorter survival and may serve as an adverse prognostic biomarker (Hussan et al., 2017). With the technology of the microbiome development, a large number of significant bacteria have been increasingly discovered. These include Proteobacteria, Bacteroidetes, Staphylococcaceae, *Alistipes*, Coriobacteriaceae, and Methanobacteriales. However, some bacteria showed diminished prevalence in CRC, including Firmicutes, *Lactobacillus*, *Faecalibacterium*, *Rumen Bacterium*, and *Bifidobacterium* (Borges-Canha et al., 2015; Gagniere et al., 2016; Hussan et al., 2017). Despite extensive research on intestinal bacteria in patients with CRC, previous investigations have not rigorously addressed possible differences in the intestinal microflora between anatomical sites. To date, little is known about the characteristics of gut microbiota alterations between proximal and distal CRCs.

In this study, we hypothesized that there would be differences in the compositions of the gut microbiome between patients with proximal colon cancer and those with distal CRC. To that end, we utilized 16S rRNA gene sequencing to compare tissue microbiota communities from patients with proximal CRC and distal CRC in a clinical cohort and to examine whether the gut microbiome differences were related with clinical features and survival outcomes. Elucidating such potential differences is important in finding new potential therapeutic strategies and advancing personalized therapies for CRC in the future.

## Materials and Methods

### Study Population and Samples

The participants enrolled in this study were Chinese patients from Wuhan Union Hospital of Tongji Medical College, Huazhong University of Science and Technology, Central China. Samples and data (demographics and clinicopathological) of the participants were collected from hospital electronic medical records. The inclusion criteria for tissue sample selection were the following: (1) patients diagnosed with primary CRC and had undergone surgical treatment; and (2) availability of tumor and adjacent normal tissues. Considering the colorectal continuum model (Papagiorgis, 2013), colon and rectal cancer cases were both included. Proximal CRC was defined as tumors from the cecum to the splenic flexure, while distal CRC was defined as tumors from the descending colon to the rectum (Lee et al., 2015; Tejpar et al., 2017). Patients treated with chemotherapy, radiotherapy, or antibiotics before surgery were excluded from this study.

A total of 329 matched samples (329 tumor tissue samples and 329 adjacent non-tumor tissue samples) were collected. Samples of paired tumor and normal mucosa tissue were fixed in formalin and embedded in paraffin (FFPE). Five serial cuts of 5 μm per sample were placed in sterile microtubes and then stored at room temperature until use for 16S rRNA MiSeq sequencing. All subjects provided written informed consent before they participated in the study. Approval of this study was obtained from the Ethics Committee of Tongji Medical College of Huazhong University of Science and Technology (Approval No. 2014-041).

### DNA Extraction, Polymerase Chain Reaction Amplification, and MiSeq Sequencing

Genomic DNA was extracted from CRC tissue and matched normal tissue samples using the Omega Mag-Bind Soil DNA Kit (Omega Bio-Tek, Norcross, GA, United States). NanoDrop (Thermo Fisher Scientific, Waltham, MA, United States) was performed for DNA quantification. The size of DNA was checked by 0.8 agarose gel electrophoresis. All extracted DNA samples were immediately stored at −80°C until further processing.

The extracted DNA was amplified by employing a series of primers targeting the V3–V4 variable region of 16S rRNA gene. The forward primer was 5′-ACTCCTACGGGAGGCAGCA-3′, and the reverse primer was 5′-GGACTACHVGGGTWTCTAAT-3′. The specific DNA barcode sequences for species identification were attached immediately after the adapter. Polymerase chain reaction (PCR) amplification was performed in a 25-μl reaction system containing 5 μl of 5 × reaction buffer, 5 μl of 5 × GC buffer, 2 μl of dNTP (2.5 mM), 1 μl of forward primer (10 μM), 1 μl of reverse primer (10 μM), 8.75 μl of ddH_2_O, 0.25 μl of Q5 DNA polymerase (NEB, M0491L), and 2 μl of DNA template. PCR was conducted in an Applied Biosystems^®^ 2720 PCR amplification instrument (Applied Biosystems Instruments, Thermo Fisher Scientific, Waltham, MA, United States) under the following conditions: initial denaturation for 2 min at 98°C; 30 cycles of 15 s at 98°C, 30 s at 55°C, and 30 s at 72°C; and final extension for 5 min at 72°C.

The PCR products were detected using two agarose gel electrophoresis. Amplicons were purified again and quantified using the Quant-iT PicoGreen dsDNA Assay Kit (Microplate reader, BioTek, FLx800, Winooski, VT, United States). All purified amplicons for each sample were mixed. The DNA library was constructed according to the TruSeq Nano DNA LT Library Prep Kit, and sequencing was performed on the Illumina MiSeqPE250.

### Sequencing Data Processing

Raw sequencing data were filtered and quality assessed; and subsequently, the query sequence was removed. The data processing then proceeded as follows: (1) raw sequencing data performed on the Illumina MiSeq platform were saved in FASTQ format, and paired-end sequencing data were filtered in a sliding window method: (a) the window size was 10 bp, and the step size was 1 bp; (b) moving from the first base position at the 5′ end; (c) the average sequencing accuracy of bases was ≥99; (d) the truncated sequence length was ≥150 bp; and (e) ambiguous base (N) was not allowed. (2) Paired-end sequenced reads of each library were overlapped by FLASH version 1.2.7^1^ (Magoè and Salzberg, 2011): (a) the overlapping base length of the two sequences Read 1 and Read 2 ≥10 bp; and (b) the mismatch base number was less than 10 of the length of the overlapping base. (3) Incorrect sequences were identified using QIIME software (Quantitative Insights Into Microbial Ecology, v1.8.0^2^) (Caporaso et al., 2010): (a) sequence length ≥160 bp; (b) no ambiguous base (N); (c) sequences with 5′ primer mismatch base numbers >1 were not allowed; and (d) sequences containing consecutive identical base numbers >8 were not allowed. (4) Chimeric sequences were detected and removed through USEARCH (v5.2.236^3^) and QIIME software (v1.8.0, see text footnote 2).

### Operational Taxonomic Unit Clustering and Taxonomy Annotation

The high-quality sequences were clustered into operational taxonomic units (OTUs) at 97 sequence similarity using USEARCH software and by the UPARSE-OTU algorithm. The sequences with the highest abundance in each OTU were selected as the representative sequences of each OTU. The representative sequence was assigned taxonomically using QIIME software by searching against the Greengenes database (Release 13.8^4^) (DeSantis et al., 2006) with default parameters. Later, an original OTU composition table was created to record the OTU abundance for each sample and the taxonomic classification for each OTU. OTUs with abundance <0.001 of the total sequences in all samples were discarded (Bokulich et al., 2013).

### Statistical Analysis

Chi-square tests and *t*-tests were used to compare demographics and tumor characteristics between patients with proximal and distal CRCs. The richness and evenness of the species were performed by R software and were represented on a rank abundance curve. Microbial alpha diversity was analyzed by sampling-based OTU table. Microbial alpha diversity was presented by Chao1, ACE, Shannon, and Simpson diversity indices (Paul et al., 2015), which were calculated using the Wilcoxon rank sum test. *t*-Test was used to compare alpha diversity and tumor characteristics in patients with proximal and distal CRCs. For beta diversity, permutational multivariate analysis of variance (PERMANOVA) was used to assess microbial community structure differences between two groups. The principal coordinates analysis (PCoA) was conducted by weighted UniFrac and unweighted UniFrac distances. For microbial taxonomic composition, we performed Metastats method to compare microbial differences between the proximal and distal tumor tissues (White et al., 2009).^5^ The expression level of Fusobacteria was categorized into “high” and “low” using the median value as cut-off point, while samples with undetectable Fusobacteria expression were categorized as negative. Chi-square tests, Fisher’s exact test, and *t*-test were used to compare the relationship between Fusobacteria and tumor characteristics. The Kaplan–Meier method was used to analyze the relationship between differential flora and OS in the patients with proximal and distal CRCs.

## Results

### Demographic Characteristics of Patients With Proximal and Distal Colorectal Cancer

**FIGURE 1:**
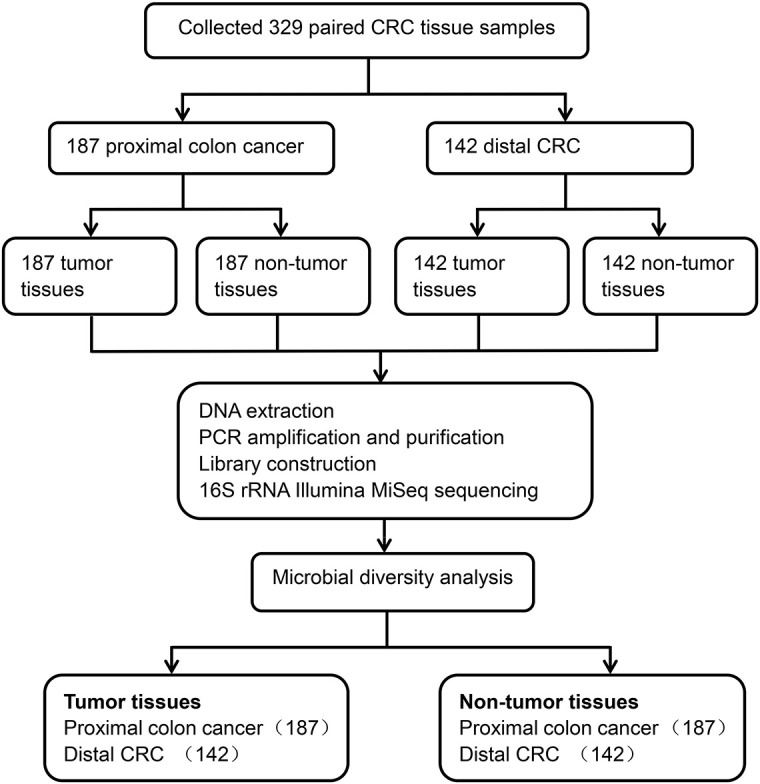
Study design and flow diagram.

A total of 329 CRC matched tumor tissues and adjacent non-tumor tissues from Chinese patients were collected. After a strict pathological diagnosis, 187 paired proximal colon cancer samples and 142 paired distal CRC tissue samples were included in this study. These samples consisted of tumor tissues and adjacent non-tumor tissues. The microbial diversity of all tissue samples collected was detected by 16S rRNA Illumina MiSeq sequencing. Microbial community analysis was performed between the proximal and distal tumor tissues as well as between the proximal and distal non-tumor tissues (Figure 1).

The clinical characteristics of patients with proximal and distal CRCs are as shown in Table 1. There were significant differences in tumor diameter between patients with proximal colon cancer and patients with distal CRC (*p* < 0.001). The tumor diameters from patients with proximal and those with distal CRC were 5.4 cm (SD, 2.3 cm) and 3.9 cm (SD, 1.7 cm), respectively. It was found that there were no significant differences in other variables, such as age, gender, or tumor differentiation between the two groups (all *p* > 0.05).

**TABLE 1:** Clinical characteristics of patients with proximal and distal CRCs.

**Characteristics**	**Total *n* = 329 (%)**	**Proximal CRC *n* = 187(%)**	**Distal CRC *n* = 142(%)**	***p*-Value**
Age (years)	57.16 ± 13.14	57.65 ± 13.85	56.53 ± 12.12	0.446
Gender				0.058
Female	140 (43)	88 (47)	52 (37)	
Male	189 (57)	99 (53)	90 (63)	
Tumor diameter (cm)	4.8 ± 2.2	5.4 ± 2.3	3.9 ± 1.7	<0.001
Tumor location				
Cecum	42 (13)	42 (23)	–	
Ascending colon	66 (20)	66 (35)	–	
Hepatic flexure	41 (12)	41 (22)	–	
Transverse colon	30 (9)	30 (16)	–	
Splenic flexure	8 (2)	8 (4)	–	
Descending colon	36 (11)	–	36 (25)	
Sigmoid colon	51 (16)	–	51 (36)	
Rectum	55 (17)	–	55 (39)	
Differentiation				0.620
Well to moderate	244 (83)	135 (82)	109 (84)	
Poor	49 (17)	29 (18)	20 (16)	

*There were 42 cases of proximal colon cancer in the cecum, 66 in the ascending colon, 41 in hepatic flexure, 30 in transverse colon, and 8 in splenic flexure. There were 36 cases of distal CRC in the descending colon, 51 in the sigmoid colon, and 55 in the rectum. The categorical and continuous variables were analyzed by Chi-square tests and two independent samples *t*-tests.*

*CRC, colorectal cancer.*

### Estimation of Sequencing Depth

**FIGURE 2:**
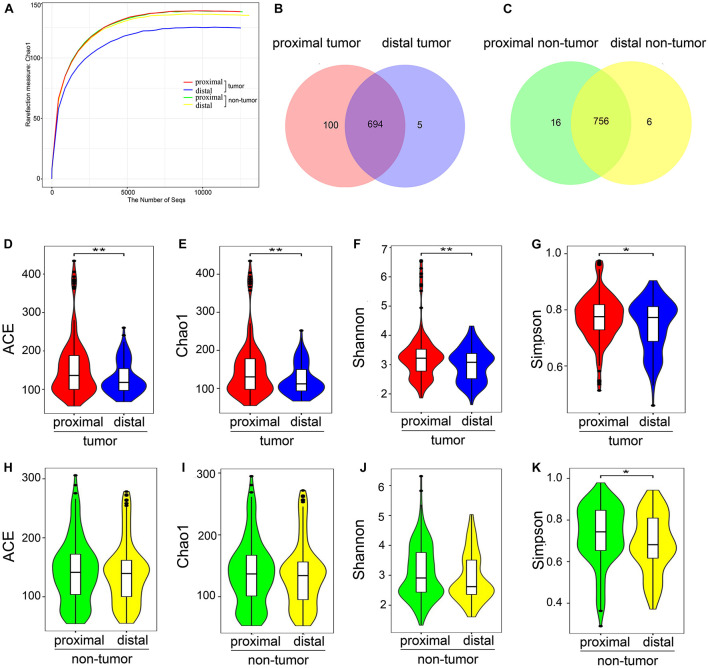
Microbial community richness and diversity in proximal and distal CRC tumor tissues. **(A)** Rarefaction curves between the number of sequences and estimated richness by Chao1. The sequencing depth basically approached saturation in all samples. **(B)** Venn diagrams. The Venn diagrams represent the shared and unique taxa among the different tissues. One hundred of 799 OTUs were unique for proximal tumor tissues compared with distal tumor tissues, while only 16 of 778 OTUs were unique for proximal non-tumor tissues compared with distal non-tumor tissues **(C)**. The microbial diversity, as estimated by the ACE index **(D)**, Chao1 index **(E)**, Shannon index **(F)**, and Simpson index **(G)**, was significantly higher in proximal tumor tissues than in distal tumor tissues (*p* = 0.0062, 0.0058, 0.0074, and 0.0125, respectively). There were no significant differences in ACE **(H)**, Chao1 **(I)**, and Shannon indices **(J)**, between proximal and distal non-tumor tissues (*p* = 0.4764, 0.4852, and 0.1218, respectively) except the Simpson index **(K)** (*p* = 0.0125); CRC, colorectal cancer; OTUs, operational taxonomic units. *p < 0.05; **p < 0.01.

A dataset of 23,220,738 high-quality sequences was obtained from 329 pairs of tissue samples. It was found that each of these samples had an average length of 430 bp and 35,389 reads. Rarefaction curves showed that the sequencing depth basically approached saturation in all samples (Figure 2A). Moreover, a Venn diagram illustrating the relationship between the groups showed that 100 of 799 OTUs were unique for proximal tumor tissues compared with distal tumor tissues (Figure 2B). On the other hand, the Venn diagram also show that only 16 of 778 OTUs were unique for proximal non-tumor tissues compared with distal non-tumor tissues (Figure 2C).

### Increased Gut Microbial Alpha Diversity in the Proximal Colorectal Cancer Group

To estimate microbial community richness and diversity within the samples, the alpha diversity indices, including ACE, Chao1, Shannon, and Simpson, were analyzed in different tissues. It was found that there were significant differences between the proximal and distal tumor tissues in ACE, Chao1, Shannon, and Simpson indices (*p* = 0.0062, 0.0058, 0.0074, and 0.0331, respectively) (Figures 2D–G). However, there were no significant differences in ACE, Chao1, and Shannon indices between the proximal and distal non-tumor tissues (*p* = 0.4764, 0.4852, and 0.1218, respectively) (Figures 2H–J) except in the Simpson index (*p* = 0.0125) (Figure 2K). The Simpson index indicated that the proximal colorectal tumor samples showed a larger variation in α-diversity values than distal CRC, whereas the non-colorectal samples had more constant values.

### Microbial Diversity Association With Tumor Clinical Parameters and Specific Microflora

A correlation analysis was performed to determine whether microbial alpha diversity was associated with clinical parameters in the proximal and distal CRC tumor tissues. It indicated that the association between the alpha diversity of the Shannon index and clinical characteristic (tumor clinical parameters tumor diameter, tumor pT stage, depth of tumor invasion, lymph node metastasis, and distant metastasis) was not statistically significant in both the proximal and distal tumor tissues (*p* > 0.05, Table 2). This study also assessed the relationship between microbial alpha diversity with the Shannon index and median overall survival of patients with CRC. However, we did not find a significant association between the alpha diversity and survival of patients with CRC (Supplementary Figure 1A). It was found that these relationships did not achieve statistical significance when analysis was performed within proximal and distal CRCs separately (Supplementary Figures 1B,C).

**TABLE 2:** Associations of alpha diversity in proximal and distal CRC tumor tissues with tumor characteristics.

**Characteristics**	**Alpha diversity (Shannon index)**			
	**Proximal tumor (*n* = 187)**	***p*-Value**	**Distal tumor (*n* = 142)**	***p*-Value**
Tumor diameter (cm)		0.519		0.906
<2	3.25		3.09	
2–5	3.36		2.30	
≥5	3.24		2.99	
pT stage		0.150		0.569
T1	2.91		2.90	
T2	3.75		2.82	
T3	3.33		3.06	
T4	3.11		2.90	
pN stage		0.401		0.065
N0	3.27		3.11	
N1–2	3.29		2.91	
Distant metastasis		0.674		0.580
M0	3.33		3.01	
M1	3.13		2.96	
Fusobacteria		0.000***		0.120
High	3.11		2.98	
Low/negative	4.66		3.28	
Acidobacteria		0.564		0.206
High	3.19		2.98	
Low/negative	3.32		3.01	
Firmicutes		0.000***		0.843
High	4.05		3.06	
Low/negative	2.97		2.99	
Bacteroidetes		0.071		0.000***
High	3.08		3.78	
Low/negative	3.34		2.86	
Proteobacteria		0.856		0.007**
High	3.29		2.84	
Low/negative	3.26		3.68	

*We defined the level of microflora (high and low/negative) based on the average abundance of microbiota in tumor tissues.*

*pT, depth of tumor invasion; M, distant metastasis of primary tumor.*

**p < 0.01; ***p ≤ 0.001.

The association between microbial alpha diversity and specific microflora was further investigated in correlation analysis (Table 2). In proximal tumor tissues, the abundance of Fusobacteria showed an inverse correlation with alpha diversity, while the enrichment of Firmicutes showed a positive correlation with alpha diversity (both *p* < 0.0001). On the other hand, in distal tumor tissues, the abundance of Proteobacteria was inversely correlated with alpha diversity, while the abundance of Bacteroidetes was associated with higher alpha diversity (both *p* < 0.0001).

### Microbial Communities in Composition in Proximal and Distal Colorectal Cancer

Beta diversity was assessed using PERMANOVA to determine the differences in microbial compositions between sub-anatomical samples. Both unweighted and weighted UniFrac distance indicated that the proximal and distal CRC samples had a statistically significant difference on microbial compositions (*p* = 0.002, Table 3). For non-tumor tissues, unweighted UniFrac distance showed a significant difference (*p* = 0.033, Table 3) between the normal proximal and distal intestinal tissues. However, the weighted UniFrac analysis indicated that there was no significant difference between the two normal groups (*p* = 0.209, Table 3).

**TABLE 3:** Beta diversity assessed by weighted and unweighted UniFrac distances.

**Metric**	**Model**	***F***	***R* ^2^**	***p*-Value**
UniFrac distances (unweighted)				
	Proximal vs. distal (tumor)	4.833	0.015	0.002**
	Proximal vs. distal (non-tumor)	2.021	0.006	0.033*
UniFrac distances (weighted)				
	Proximal vs. distal (tumor)	6.302	0.019	0.002**
	Proximal vs. distal (non-tumor)	1.454	0.004	0.209

*Weighted and unweighted UniFrac distances between different samples.*

*p < 0.05; **p < 0.01; ***p ≤ 0.001.

**FIGURE 3:**
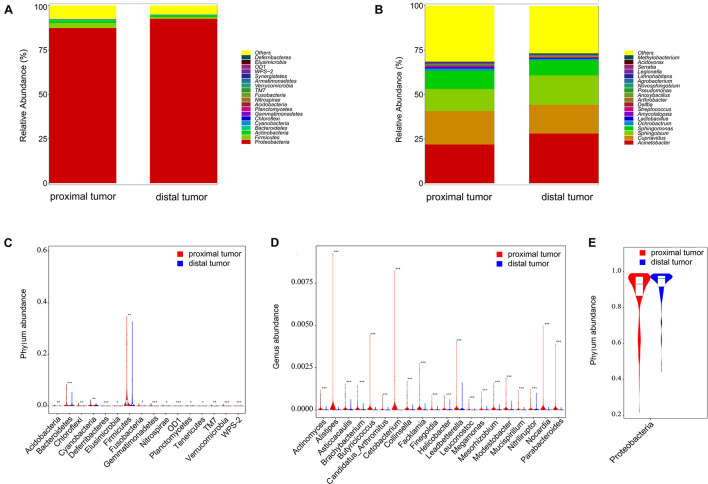
Profiles of the microbial compositions and differential communities at the phylum and genus levels. Microbiota compositions at the phylum level **(A)** and genus level **(B)** between proximal and distal tumor tissues. The enriched microbial communities in proximal tumor tissues vs. distal tumor tissues, at the phylum level **(C)** and genus level **(D)**. The enriched microbial community in distal tumor tissues vs. proximal tumor tissues at the phylum level **(E)**. ^∗^*p* < 0.05; ^∗∗^*p* < 0.01; ^∗∗∗^*p* < 0.001.

At the phylum level, the four most relatively abundant phyla in the 329 CRC tissues were Proteobacteria, Firmicutes, Actinobacteria, and Bacteroidetes, accounting for 92% of the total community (Figure 3A). At the genus level, the four dominant genera in the 329 CRC tissues were *Acinetobacter*, *Cupriavidus*, *Sphingobium*, and *Sphingomonas* (Figure 3B). To further compare microbial community differences between the proximal and distal tumor tissues, we conducted the Metastats comparison. As shown in Figure 3C, 16 phyla including Acidobacteria, Bacteroidetes, Chloroflexi, Cyanobacteria, Deferribacteres, Elusimicrobia, Firmicutes, and Fusobacteria were enriched in proximal tumor tissues compared with distal tumor tissues (*p* < 0.05). Correspondingly, the genera *Actinomyces*, *AlistiPes*, and *Helicobacter* were increased in proximal tumor tissues compared with distal tumor tissues (*p* < 0.05, Figure 3D). In addition, Proteobacteria were enriched in distal tumor tissues compared with proximal tumor tissues (*p* < 0.05, Figure 3E). These results show that the composition of microbial community differs in proximal and distal CRCs, but a nearly constant community is maintained in normal proximal and distal colorectal tissues.

### Association of Fusobacteria Status With Clinical Characteristics and Outcomes

This study also tested the association of the status of Fusobacteria with the clinical characteristics of the 329 CRC patients. This was because *Fusobacterium* species are important in the development of CRC. It was revealed that the status of Fusobacteria was associated with the age of the patients (*p* = 0.019, Table 4) and tumor diameter (*p* = 0.046, Table 4). In addition, the results of this study also indicated that Fusobacteria was positivity associated with MSI status (*p* < 0.0001, Table 4). However, there was no association was found for the genetic mutations in *KRAS*, *NRAS*, *BRAF*, and *PIK3CA* (all *p* > 0.05, Table 4).

**TABLE 4:** Characteristics according to the relative abundance of Fusobacteria in CRC tissue.

**Characteristics**	**Fusobacteria – high**	**Fusobacteria – low/negative**	***p*-Value**
Age (years)	62.42 ± 13.2	56.62 ± 13.01	0.019*
Gender			0.403
Female	11 (35)	129 (42)	
Male	20 (65)	169 (58)	
Tumor diameter (cm)	5.5 ± 2.6	4.7 ± 1.4	0.046*
Differentiation			0.165
Well to moderate	21 (75)	223 (84)	
Poor	7 (25)	42 (16)	
pT stage			0.398
T1 + T2	3 (10)	14 (5)	
T3 + T4	27 (90)	248 (95)	
Lymph node metastasis			0.133
N0	20 (65)	150 (50)	
N1 + N2	11 (35)	148 (50)	
Distant metastasis			0.247
M0	27 (90)	234 (82)	
M1	3 (10)	53 (18)	
AJCC disease stage			0.057
I–II	17 (59)	112 (40)	
III–IV	12 (41)	166 (60)	
Liver metastasis			0.249
Absent	30 (97)	270 (91)	
Present	1 (3)	28 (9)	
MSI status			0.000***
MSI-high	9 (43)	20 (10)	
Non-MSI high	12 (57)	185 (90)	
*KRAS* mutation			0.268
Wild-type	8 (40)	74 (53)	
Mutant	12 (60)	65 (47)	
*NRAS* mutation			0.585
Wild-type	19 (95)	129 (93)	
Mutant	1 (5)	10 (7)	
*BRAF* mutation			0.366
Wild-type	18 (90)	131 (94)	
Mutant	2 (10)	8 (6)	
PIK3CA mutation			0.228
Wild-type	1 (50)	14 (93)	
Mutant	1 (50)	1 (7)	

*The *p*-values for categorical and continuous variables were analyzed by Chi-square test and Fisher’s exact test, and continuous variables were analyzed by two independent-samples *t*-test.*

*AJCC, American Joint Committee on Cancer; MSI, microsatellite instability; CRC, colorectal cancer.*

*p < 0.05; ***p < 0.001.

**FIGURE 4:**
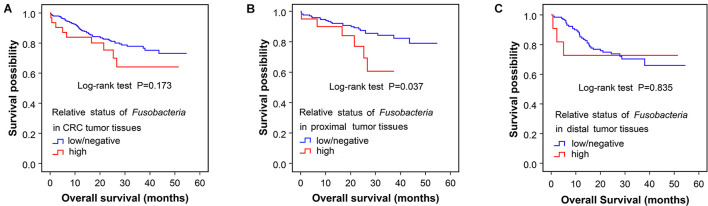
Association of Fusobacteria enriched in proximal tumor tissues with clinical overall survival. The Kaplan–Meier curves for colorectal cancer overall survival according to the relative status of Fusobacteria in CRC tumor tissues **(A)**, the relative status of Fusobacteria in proximal tumor tissues **(B)**, and the relative status of Fusobacteria in distal tumor tissues **(C)**. Cases with Fusobacteria were categorized as high or low/negative using the medium value as cutoff point. The *p*-value was calculated by the log-rank test. CRC, colorectal cancer.

Furthermore, the correlation between *Fusobacterium* status and survival outcomes in the patients with CRC was tested through the Kaplan–Meier analysis. It was revealed that the median survival time of Fusobacteria-high CRC patients was shorter than that of Fusobacteria-negative patients (Figure 4A). However, this difference was not statistically significant (*p* = 0.173). Stratification analyses of the association of *Fusobacterium* status with overall survival time in 329 CRC showed that high Fusobacteria in proximal CRC tissues was associated with significantly shorter survival time (*p* = 0.037, Figure 4B) than Fusobacteria-negative populations. However, the trend was not obvious in patients with distal CRC (*p* = 0.835, Figure 4C).

Meanwhile, the association of other enriched microbial communities in proximal tumor tissues with the survival time was also analyzed in this study. However, it was found that there was no significant association of these microbes with the survival time of patients with CRC except for Fusobacteria (*p* > 0.05) (Table 5).

**TABLE 5:** Association of microbial communities with overall survival of proximal and distal CRCs.

**Microbial communities**	**OS in proximal CRC patients (months)**			**OS in distal CRC patients (months)**		
	**High**	**Low/negative**	***p*-Value**	**High**	**Low/negative**	***p*-Value**
Firmicutes	44.7	48.3	0.185	42.8	39.5	0.356
Bacteroidetes	43.7	47.5	0.305	42.8	37.4	0.639
Proteobacteria	48.3	45.0	0.347	37.0	38.2	0.473
Cyanobacteria	46.6	45.8	0.847	38.6	37.2	0.552
Acidobacteria	46.4	47.1	0.596	37.8	41.9	0.063
Deferribacteres	46.8	46.6	0.673	45.3	45.8	0.129
Elusimicrobia	44.8	46.9	0.467	46.1	40.5	0.238
Nitrospirae	44.2	47.5	0.618	37.8	42.7	0.237
*OD1*	51.8	45.6	0.068	40.6	41.1	0.855
Planctomycetes	46.7	46.4	0.678	41.5	32.3	0.830
Tenericutes	45.7	46.8	0.881	43.4	41.3	0.789
*TM7*	46.9	46.1	0.947	46.8	40.9	0.136
Verrucomicrobia	45.3	46.6	0.791	39.8	41.3	0.991
*WPS2*	47.1	46.5	0.985	45.5	40.4	0.269
Fusobacteria	26.6	38.5	0.005**	29.5	34.7	0.789

*We define the level of microflora (high and low/negative) based on the median abundance of microbiota in tumor tissues.*

OS, overall survival; CRC, colorectal cancer. *p < 0.05; **p < 0.01.

## Discussion

This study tested the hypothesis that microbial communities for proximal and distal CRCs was heterogeneous and related to clinical features and survival outcomes. The 16S rRNA high-throughput sequencing in 329 pairs of tumor tissues and extratumoral paracancerous tissues (187 pairs of proximal CRC and 142 pairs of distal CRC) revealed different microbial alpha and beta diversities between the proximal and distal CRC tissues. However the 16S rRNA high-throughput sequencing shows relatively constant microbial diversity in the paracancerous non-cancer tissues of the proximal and distal colorectum. A comparative study of subanatomical tumor microbiota showed that proximal CRC was rich in Acidobacteria, Bacteroidetes, Chloroflexi, Cyanobacteria, Deferribacteres, Elusimicrobia, Firmicutes, and Fusobacteria, while distal CRC was rich in Proteobacteria at the phylum level. Additionally, we found that the status of Fusobacteria was associated with the age of the patients, tumor diameter, and MSI status, but not with genetic mutations in KRAS, NRAS, BRAF, and PIK3CA. Notably, Fusobacteria-high individuals had weak prognosis in proximal colon cancers, but Fusobacteria was not significantly associated with survival time in distal CRC. The results of this study suggest that significant heterogeneity in microbial diversity and composition exists in proximal colon cancers and distal CRCs. Furthermore, it is evident that Fusobacteria is an adverse prognostic factor especially in proximal colon cancer. To the best of our knowledge, this is the first study to explore the difference of the tumor microbiome on clinical outcomes between proximal colon cancers and distal CRCs.

Anatomically, CRC is divided into the splenic curvature into distal CRC (including the descending colon, sigmoid colon, and rectum) and proximal CRC (which include the cecum, and ascending and transverse colon). Epidemiological analysis shows that the incidence of proximal CRC is increasing while that of distal CRC is decreasing worldwide (Benedix et al., 2010a; Brenner et al., 2010). The differences in biological behavior of proximal and distal CRCs have been the focus of several clinical studies. Bufill (1990) first systematically expounded the differences of proximal and distal CRCs from the aspects of epidemiology, pathology, cytogenetics, molecular characteristics, and carcinogenesis mechanism and proposed that proximal and distal CRCs were two status of diseases. In recent years, more scientific data have further validated this view (Lanza et al., 1994; Iacopetta, 2002; Benedix et al., 2010b; Yang et al., 2018). However, the gut microbiota heterogeneity in CRC has not been fully delineated. Our study elucidated that proximal and distal CRCs also have intestinal microbial differences in the tumor microenvironment, and these results offered a complementary perspective to CRC heterogeneity studies. Recently, Riquelme et al. (2019) first reported that the level of alpha diversity of tumor tissue in patients with pancreatic cancer correlated with the prognosis and survival rate of the patients. Our study suggests that microbial diversity and composition are relatively constant in adjacent non-tumor tissues between the proximal and distal colorectum. However, it was suggested that for tumor tissues, the species diversity in proximal colon cancers is more abundant than distal CRCs. Additionally, further analysis did not find a potential relationship between the alpha diversity and the survival time. The most abundant phyla in the CRC tissues were Proteobacteria, Firmicutes, Actinobacteria, and Bacteroidetes. This finding is partly consistent with reports by Xu et al. (2020), who scrutinized the previous data on intestinal bacteria from patients with CRC. Interestingly, in the proximal tumor tissue, the relatively abundant phyla were Acidobacteria, Bacteroidetes, Chloroflexi, Cyanobacteria, Deferribacteres, Elusimicrobia, Firmicutes, and Fusobacteria. Only Proteobacteria was found enriched in distal tumor tissues. Although the underlying mechanism of microbial enrichment in the right colon cancer is not clear, the microenvironmental or biological factors specifically found in the proximal colon region were likely important potential influencing factors. For instance, Dejea et al. (2014) found that the bacterial biofilms in the right colon tumor were more dense as compared with those in the left colon tumor. Moreover, the present study found that the abundance of the Firmicutes had a negative correlation with the alpha diversity level, which collaborates the findings of Flemer et al. (2017). Meanwhile, it was also detected that Fusobacteria in proximal colon cancer and Proteobacteria in distal CRC were negatively correlated with the microbiota alpha diversity level.

Several studies have shown that CRC with Fusobacteria-high induced a series of specific tumor molecular events, including CIMP, MSI, and genetic mutations in KRAS, BRAF, TP53, etc. (Tahara et al., 2014; Mima et al., 2015, 2016; Yamaoka et al., 2018). Our study indicates that the abundance of Fusobacteria in CRC tumor tissues was closely related to tumor diameter, which was consistent with the report by Yamaoka et al. (2018). Additionally, it was found that the abundance of Fusobacteria in CRC tumor tissues was associated with MSI status, but not KRAS, NRAS, BRAF, and PIK3CA gene status. These results are in agreement with the findings of a United States population-based cohort study that showed that Fusobacteria was associated with *MSI* status independent of *BRAF* and *CIMP* mutation status (Mima et al., 2016). Tahara et al. (2014) also reported that the abundance of Fusobacteria was associated with MSI, CIMP, TP53, CHD7, and CHD8 mutation status but not KRAS and BRAF. However, a Japanese study reported that higher abundance of Fusobacteria in CRC tumor tissues was associated with *KRAS* mutation (Yamaoka et al., 2018). These discordant results could be due to different geographical or racial/ethnic cross study populations.

*Fusobacterium* is a non-spore-forming Gram-negative rod belonging to the family of Fusobacteriaceae (Olsen, 2014). There is emerging evidence implicating the potential involvement of *Fusobacterium* in CRC (Kostic et al., 2013; Rubinstein et al., 2013). Previous studies suggested that the abundance of *Fusobacterium* in CRC is higher than that of healthy individuals. Furthermore, the high level of *Fusobacterium* in CRC usually predicts poor prognosis. Consistently, the present study found that the CRC tissues had more abundant Fusobacteria than the paired non-tumor tissues. Two other interesting findings are also highlighted in this study. First, in CRC, the abundance of Fusobacteria in proximal tumor tissues was higher than that of the distal CRC tissues. These results corroborate the findings of Mima et al. (2016), who suggested that the proportion of *Fusobacterium*-high CRC gradually increased from the rectum to cecum, with a statistically significant linear trend along all subsites. Second, the higher abundance of Fusobacteria in proximal colon cancer is associated with shorter overall survival of patients by the Kaplan–Meier survival analysis. In distal CRC tissues, though Fusobacteria-high individuals had the least survival time than the Fusobacteria-low/negative individuals, the difference was not statistically significant. This finding has important implications in that because proximal colon cancer usually has a worse prognosis and is prone to peritoneal metastasis (Petrelli et al., 2017; Cannon and Buechler, 2019), this highly aggressive microorganism of Fusobacteria may act as one of accomplices behind the scenes. Some scholars discovered that *Fusobacterium* adhered to human epithelial cells (Han et al., 2000; Han et al., 2004), activated WNT signals (Rubinstein et al., 2013), and promoted oncogene expression (Rubinstein et al., 2013). In CRC, *Fusobacterium* facilitated carcinogenesis mainly through two toxic factors, Fap2 (Gur et al., 2015) and Fad A (Rubinstein et al., 2013). It was recently found that *Fusobacterium* promotes CRC chemoresistance by regulating autophagy and the Toll-like receptor (Yu et al., 2017). This suggests that Fusobacteria plays an important role in the development of proximal colon cancer, which may be an important focus for future prevention and treatment of proximal CRC.

The strengths of this study include that it used a relatively large sample size, integrated tumor, and patient basic information. The study also used the follow-up survival data to analyze microbial differences in distal, proximal CRC tissues, and distal and proximal paracancerous normal tissues as well as to explore the relationship of the differences in the clinical features and survival rates.

This study had some several limitations. First, routine histopathological procedures may have affected the ability of 16S rRNA sequencing to uncover the microorganisms in FFPE tissue specimens, and potential DNA contamination may exist. Although there was a possibility that the measurement errors in FFPE tissue specimens may have pushed the results to the null hypothesis, the unmeasured confounders cannot be ruled out. However, our validation study showed differences in Fusobacteria using FFPE tissue specimens, which is consistent with the previous study by Mima et al. (2016). In addition, our data on the Fusobacteria relationship with the tumor molecular characteristics of MSI and KRAS/NRAS/BRAF status were consistent with studies using frozen tissue specimens (Tahara et al., 2014) and another study with FFPE tissue specimens (Mima et al., 2016). Second, the patients who had received radiotherapy, chemotherapy, targeted therapy, or immunotherapy prior to surgery were excluded from the study because these systematic treatments may affect the detection of microorganism in surgical specimens. Third, we did not synchronously examine other specimen of microbes, such as using saliva and feces in CRC individuals. Fourth, the patients were enrolled from only a single large-scale general institution, and their data were analyzed retrospectively. Recently, a small population-based study in the United Kingdom analyzed the microbial difference in 24 anemic CRC patients receiving intravenous iron therapy (Phipps et al., 2021). It showed that the microbial diversity between the right and left subsites of CRC tumor tissues was not obvious. In fact, very few CRC patients would undergo intravenous iron therapy before surgery, and such cases are not representative of the full gamut of the disease. But whether the sample type and size, population race, or iron supplements leads to the different results is worthy of further exploration. Nevertheless, given the complicated interaction of microbiota with the host in human CRC, this retrospective study provides new data with an important reference value. However, there is a need for future prospectively designed investigations with a large sample on the gut microbiota using fresh tissue and fecal microbiota to be validated for the heterogeneity of CRC.

## Conclusion

This study demonstrates the differences in the composition and diversity of the intestinal microbial communities between the proximal and distal tumor tissues. The abundance of Fusobacteria in CRC tissue was specifically associated with age, tumor diameter, and MSI status. Moreover, Fusobacteria was a potential microbial biomarker predicting the prognosis of proximal colon cancers but not distal CRCs. The findings of this study provide a theoretical reference for more accurate understanding of intestinal microbial diversity and differences between proximal and distal CRCs. Given the potentially profound implication of the Fusobacteria on proximal colon cancers, further studies to predict the occurrence of lesions, to assess the risk of recurrence *via* Fusobacteria changes, and to design appropriate combination therapy strategies in CRC precise treatment are warranted.

## Data Availability Statement

The datasets presented in this study can be found in online repositories. The names of the repository/repositories and accession number(s) can be found in the article/Supplementary Material.

## Ethics Statement

This study was approved by the Ethics Committee of Tongji Medical College of Huazhong University of Science and Technology (No. 2014-041). The patients/participants provided their written informed consent to participate in this study.

## Author Contributions

All authors took part in design of the study protocol, collection, analysis, and interpretation of the data. HL contributed to the conception, designed the study, and critically reviewed the manuscript. MJ and FS made substantial contributions to analysis, interpretation of the data, and had the main responsibility of preparing the manuscript. JW and QF provided the assistance with the sample collection. LL provided the statistical expertise. XN and JF provided the expert skills in histopathology. TZ and KC critically reviewed the manuscript for important intellectual content. SO provided the helpful discussions and technical support. All authors have reviewed the final version of the manuscript and approve it for publication.

## Conflict of Interest

The authors declare that the research was conducted in the absence of any commercial or financial relationships that could be construed as a potential conflict of interest.

## Publisher’s Note

All claims expressed in this article are solely those of the authors and do not necessarily represent those of their affiliated organizations, or those of the publisher, the editors and the reviewers. Any product that may be evaluated in this article, or claim that may be made by its manufacturer, is not guaranteed or endorsed by the publisher.

## Footnotes

1
http://ccb.jhu.edu/software/FLASH/

2
http://qiime.org/

3
http://www.drive5.com/usearch/

4
http://greengenes.secondgenome.com/

5
http://metastats.cbcb.umd.edu/

## References

[B1] Abdulamir A. S. Hafidh R. R. Mahdi L. K. Al-jeboori T. Abubaker F. (2009). Investigation into the controversial association of Streptococcus gallolyticus with colorectal cancer and adenoma. *BMC Cancer* 9:403. 10.1186/1471-2407-9-403 19925668 PMC2785837

[B2] Benedix F. Kube R. Meyer F. Schmidt U. Gastinger I. Lippert H. (2010a). Comparison of 17,641 patients with right- and left-sided colon cancer: differences in epidemiology, perioperative course, histology, and survival. *Dis. Colon. Rectum.* 53:1. 10.1007/DCR.0b013e3181c703a4 20010352

[B3] Benedix F. Meyer F. Kube R. Gastinger I. Lippert H. (2010b). [Right- and left-sided colonic cancer - different tumour entities]. *Zentralbl. Chir.* 135:4. 10.1055/s-0030-1247471 20806133

[B4] Bokulich N. A. Subramanian S. Faith J. J. Gevers D. Gordon J. I. Knight R. (2013). Quality-filtering vastly improves diversity estimates from Illumina amplicon sequencing. *Nat Methods* 10:1. 10.1038/nmeth.2276 23202435 PMC3531572

[B5] Borges-Canha M. Portela-Cidade J. P. Dinis-Ribeiro M. Leite-Moreira A. F. Pimentel-Nunes P. (2015). Role of colonic microbiota in colorectal carcinogenesis: a systematic review. *Rev. Esp. Enferm. Dig.* 107:11. 10.17235/reed.2015.3830/2015 26541655

[B6] Bray F. Ferlay J. Soerjomataram I. Siegel R. L. Torre L. A. Jemal A. (2018). Global cancer statistics 2018: gLOBOCAN estimates of incidence and mortality worldwide for 36 cancers in 185 countries. *CA Cancer J. Clin.* 68:6. 10.3322/caac.21492 30207593

[B7] Brennan C. A. Garrett W. S. (2016). Gut Microbiota, Inflammation, and Colorectal Cancer. *Annu. Rev. Microbiol.* 70 395–411. 27607555 10.1146/annurev-micro-102215-095513 PMC5541233

[B8] Brenner H. Hoffmeister M. Arndt V. Stegmaier C. Altenhofen L. Haug U. (2010). Protection from right- and left-sided colorectal neoplasms after colonoscopy: population-based study. *J. Natl. Cancer Inst.* 102:2. 10.1093/jnci/djp436 20042716

[B9] Bufill J. A. (1990). Colorectal cancer: evidence for distinct genetic categories based on proximal or distal tumor location. *Ann. Intern. Med.* 113:10. 10.7326/0003-4819-113-10-779 2240880

[B10] Bullman S. Pedamallu C. S. Sicinska E. Clancy T. E. Zhang X. Cai D. (2017). Analysis of Fusobacterium persistence and antibiotic response in colorectal cancer. *Science* 358:6369. 10.1126/science.aal5240 29170280 PMC5823247

[B11] Cannon E. Buechler S. (2019). Colon Cancer Tumor Location Defined by Gene Expression May Disagree With Anatomic Tumor Location. *Clin. Colorectal. Cancer* 18 149–158. 10.1016/j.clcc.2019.02.002 30853326

[B12] Caporaso J. G. Kuczynski J. Stombaugh J. Bittinger K. Bushman F. D. Costello E. K. (2010). QIIME allows analysis of high-throughput community sequencing data. *Nat. Methods* 7:5. 10.1038/nmeth.f.303 20383131 PMC3156573

[B13] Dejea C. M. Wick E. C. Hechenbleikner E. M. White J. R. Mark Welch J. L. Rossetti B. J. (2014). Microbiota organization is a distinct feature of proximal colorectal cancers. *Proc. Natl. Acad. Sci. U. S. A.* 111:51. 10.1073/pnas.1406199111 25489084 PMC4280621

[B14] DeSantis T. Z. Hugenholtz P. Larsen N. Rojas M. Brodie E. L. Keller K. (2006). Greengenes, a chimera-checked 16S rRNA gene database and workbench compatible with ARB. *Appl. Environ. Microbiol.* 72:7. 10.1128/AEM.03006-05 16820507 PMC1489311

[B15] Eckburg P. B. Bik E. M. Bernstein C. N. Purdom E. Dethlefsen L. Sargent M. (2005). Diversity of the human intestinal microbial flora. *Science* 308:5728. 10.1126/science.1110591 PMC1395357 15831718

[B16] Flemer B. Lynch D. B. Brown J. M. Jeffery I. B. Ryan F. J. Claesson M. J. (2017). Tumour-associated and non-tumour-associated microbiota in colorectal cancer. *Gut* 66:4. 10.1136/gutjnl-2015-309595 26992426 PMC5529966

[B17] Gagniere J. Raisch J. Veziant J. Barnich N. Bonnet R. Buc E. (2016). Gut microbiota imbalance and colorectal cancer. *World J. Gastroenterol.* 22:2. 10.3748/wjg.v22.i2.501 26811603 PMC4716055

[B18] Guinney J. Dienstmann R. Wang X. de Reynies A. Schlicker A. Soneson C. (2015). The consensus molecular subtypes of colorectal cancer. *Nat. Med.* 21:11. 10.1038/nm.3967 26457759 PMC4636487

[B19] Gur C. Ibrahim Y. Isaacson B. Yamin R. Abed J. Gamliel M. (2015). Binding of the Fap2 protein of *Fusobacterium nucleatum* to human inhibitory receptor TIGIT protects tumors from immune cell attack. *Immunity* 42:2. 10.1016/j.immuni.2015.01.010 PMC4361732 25680274

[B20] Han Y. W. Redline R. W. Li M. Yin L. Hill G. B. McCormick T. S. (2004). *Fusobacterium nucleatum* induces premature and term stillbirths in pregnant mice: implication of oral bacteria in preterm birth. *Infect. Immun.* 72:4. 10.1128/IAI.72.4.2272-2279.2004 15039352 PMC375172

[B21] Han Y. W. Shi W. Huang G. T. Kinder Haake S. Park N. H. Kuramitsu H. (2000). Interactions between periodontal bacteria and human oral epithelial cells: *fusobacterium nucleatum* adheres to and invades epithelial cells. *Infect Immun.* 68:6. 10.1128/IAI.68.6.3140-3146.2000 10816455 PMC97547

[B22] Hussan H. Clinton S. K. Roberts K. Bailey M. T. (2017). Fusobacterium’s link to colorectal neoplasia sequenced: a systematic review and future insights. *World J. Gastroenterol.* 23:48. 10.3748/wjg.v23.i48.8626 29358871 PMC5752723

[B23] Iacopetta B. (2002). Are there two sides to colorectal cancer? *Int. J. Cancer* 101:5. 10.1002/ijc.10635 12216066

[B24] Kostic A. D. Chun E. Robertson L. Glickman J. N. Gallini C. A. Michaud M. (2013). *Fusobacterium nucleatum* potentiates intestinal tumorigenesis and modulates the tumor-immune microenvironment. *Cell Host. Microbe.* 14:2. 10.1016/j.chom.2013.07.007 PMC3772512 23954159

[B25] Lanza G. Jr. Maestri I. Ballotta M. R. Dubini A. Cavazzini L. (1994). Relationship of nuclear DNA content to clinicopathologic features in colorectal cancer. *Mod. Pathol.* 7:2. 8008736

[B26] Larabi A. Barnich N. Nguyen H. T. T. (2019). New insights into the interplay between autophagy, gut microbiota and inflammatory responses in IBD. *Autophagy* 16 38–51. 10.1080/15548627.2019.1635384 31286804 PMC6984609

[B27] Lee G. H. Malietzis G. Askari A. Bernardo D. Al-Hassi H. O. Clark S. K. (2015). Is right-sided colon cancer different to left-sided colorectal cancer? - a systematic review. *Eur. J. Surg. Oncol.* 41:3. 10.1016/j.ejso.2014.11.001 25468456

[B28] Lee M. S. Menter D. G. Kopetz S. (2017). Right Versus Left Colon Cancer Biology: integrating the Consensus Molecular Subtypes. *J. Natl. Compr. Canc Netw.* 15:3. 10.6004/jnccn.2017.0038 28275039

[B29] Ley R. E. Peterson D. A. Gordon J. I. (2006). Ecological and evolutionary forces shaping microbial diversity in the human intestine. *Cell* 124:4. 10.1016/j.cell.2006.02.017 16497592

[B30] Loree J. M. Pereira A. A. L. Lam M. Willauer A. N. Raghav K. Dasari A. (2018). Classifying Colorectal Cancer by Tumor Location Rather than Sidedness Highlights a Continuum in Mutation Profiles and Consensus Molecular Subtypes. *Clin. Cancer Res.* 24:5. 10.1158/1078-0432.CCR-17-2484 29180604 PMC5844818

[B31] Magoè T. Salzberg S. L. (2011). FLASH: fast length adjustment of short reads to improve genome assemblies. *Bioinformatics* 27:21. 10.1093/bioinformatics/btr507 21903629 PMC3198573

[B32] Mima K. Cao Y. Chan A. T. Qian Z. R. Nowak J. A. Masugi Y. (2016). *Fusobacterium nucleatum* in Colorectal Carcinoma Tissue According to Tumor Location. *Clin. Transl. Gastroenterol.* 7:11. 10.1038/ctg.2016.53 27811909 PMC5543402

[B33] Mima K. Sukawa Y. Nishihara R. Qian Z. R. Yamauchi M. Inamura K. (2015). *Fusobacterium nucleatum* and T Cells in Colorectal Carcinoma. *JAMA Oncol.* 1:5. 10.1001/jamaoncol.2015.1377 26181352 PMC4537376

[B34] Missiaglia E. Jacobs B. D’Ario G. Di Narzo A. F. Soneson C. Budinska E. (2014). Distal and proximal colon cancers differ in terms of molecular, pathological, and clinical features. *Ann. Oncol.* 25:10. 10.1093/annonc/mdu275 25057166

[B35] Olsen I. (2014). “The Family Fusobacteriaceae” in *The Prokaryotes: firmicutes and Tenericutes.* eds Rosenberg E. DeLong E. F. Lory S. Stackebrandt E. Thompson F. (Berlin: Springer). 109–132. 10.1007/978-3-642-30120-9_213

[B36] Papagiorgis P. (2013). Colorectal cancer: dichotomous or continuum model? Perhaps, a combination of both. *Gut* 62:10. 10.1136/gutjnl-2013-305209 23749607

[B37] Paul D. Kumbhare S. V. Mhatre S. S. Chowdhury S. P. Shetty S. A. Marathe N. P. (2015). Exploration of Microbial Diversity and Community Structure of Lonar Lake: the Only Hypersaline Meteorite Crater Lake within Basalt Rock. *Front. Microbiol.* 6:1553. 10.3389/fmicb.2015.01553 26834712 PMC4722114

[B38] Petrelli F. Tomasello G. Borgonovo K. Ghidini M. Turati L. Dallera P. (2017). Prognostic Survival Associated With Left-Sided vs Right-Sided Colon Cancer: a Systematic Review and Meta-analysis. *JAMA Oncol.* 3:2. 10.1001/jamaoncol.2016.4227 27787550

[B39] Phipps O. Quraishi M. N. Dickson E. A. Steed H. Kumar A. Acheson A. G. (2021). Differences in the On- and Off-Tumor Microbiota between Right- and Left-Sided Colorectal Cancer. *Microorganisms* 9:5. 10.3390/microorganisms9051108 34065545 PMC8160982

[B40] Riquelme E. Zhang Y. Zhang L. Montiel M. Zoltan M. Dong W. (2019). Tumor Microbiome Diversity and Composition Influence Pancreatic Cancer Outcomes. *Cell* 178:4. 10.1016/j.cell.2019.07.008 31398337 PMC7288240

[B41] Rubinstein M. R. Wang X. Liu W. Hao Y. Cai G. Han Y. W. (2013). *Fusobacterium nucleatum* promotes colorectal carcinogenesis by modulating E-cadherin/beta-catenin signaling via its FadA adhesin. *Cell Host. Microbe.* 14:2. 10.1016/j.chom.2013.07.012 23954158 PMC3770529

[B42] Sokic-Milutinovic A. Alempijevic T. Milosavljevic T. (2015). Role of *Helicobacter* pylori infection in gastric carcinogenesis: current knowledge and future directions. *World J. Gastroenterol.* 21:41. 10.3748/wjg.v21.i41.11654 26556993 PMC4631967

[B43] Tahara T. Yamamoto E. Suzuki H. Maruyama R. Chung W. Garriga J. (2014). Fusobacterium in colonic flora and molecular features of colorectal carcinoma. *Cancer Res.* 74:5. 10.1158/0008-5472.CAN-13-1865 24385213 PMC4396185

[B44] Tejpar S. Stintzing S. Ciardiello F. Tabernero J. Van Cutsem E. Beier F. (2017). Prognostic and Predictive Relevance of Primary Tumor Location in Patients With RAS Wild-Type Metastatic Colorectal Cancer: retrospective Analyses of the CRYSTAL and FIRE-3 Trials. *JAMA Oncol.* 3:2. 10.1001/jamaoncol.2016.3797 27722750 PMC7505121

[B45] Thomas A. M. Manghi P. Asnicar F. Pasolli E. Armanini F. Zolfo M. (2019). Metagenomic analysis of colorectal cancer datasets identifies cross-cohort microbial diagnostic signatures and a link with choline degradation. *Nat. Med.* 25:4. 10.1038/s41591-019-0405-7 PMC9533319 30936548

[B46] Tjalsma H. Boleij A. Marchesi J. R. Dutilh B. E. (2012). A bacterial driver-passenger model for colorectal cancer: beyond the usual suspects. *Nat. Rev. Microbiol.* 10:8. 10.1038/nrmicro2819 22728587

[B47] White J. R. Nagarajan N. Pop M. (2009). Statistical methods for detecting differentially abundant features in clinical metagenomic samples. *PLoS Comput. Biol.* 5:4. 10.1371/journal.pcbi.1000352 19360128 PMC2661018

[B48] Xu S. Yin W. Zhang Y. Lv Q. Yang Y. He J. (2020). Foes or Friends? Bacteria Enriched in the Tumor Microenvironment of Colorectal Cancer. *Cancers* 12:2. 10.3390/cancers12020372 32041122 PMC7072156

[B49] Yamaoka Y. Suehiro Y. Hashimoto S. Hoshida T. Fujimoto M. Watanabe M. (2018). *Fusobacterium nucleatum* as a prognostic marker of colorectal cancer in a Japanese population. *J. Gastroenterol.* 53:4. 10.1007/s00535-017-1382-6 28823057

[B50] Yang S. Y. Cho M. S. Kim N. K. (2018). Difference between right-sided and left-sided colorectal cancers: from embryology to molecular subtype. *Expert Rev. Anticancer Ther.* 18:4. 10.1080/14737140.2018.1442217 29458272

[B51] Yu T. Guo F. Yu Y. Sun T. Ma D. Han J. (2017). *Fusobacterium nucleatum* Promotes Chemoresistance to Colorectal Cancer by Modulating Autophagy. *Cell* 170:3. 10.1016/j.cell.2017.07.008 28753429 PMC5767127

[B52] Zumkeller N. Brenner H. Zwahlen M. Rothenbacher D. (2006). *Helicobacter* pylori infection and colorectal cancer risk: a meta-analysis. *Helicobacter* 11:2. 10.1111/j.1523-5378.2006.00381.x 16579836

