# Supplementary material for: Tumor-Associated Microbiota in Proximal and Distal Colorectal Cancer and Their Relationships With Clinical Outcomes
Source: Front Microbiol. 2021 Sep 28;12:727937. doi: 10.3389/fmicb.2021.727937 (PMC8506159; doi:10.3389/fmicb.2021.727937)
Supplement: Supplementary file 1 [file Data_Sheet_1.docx]

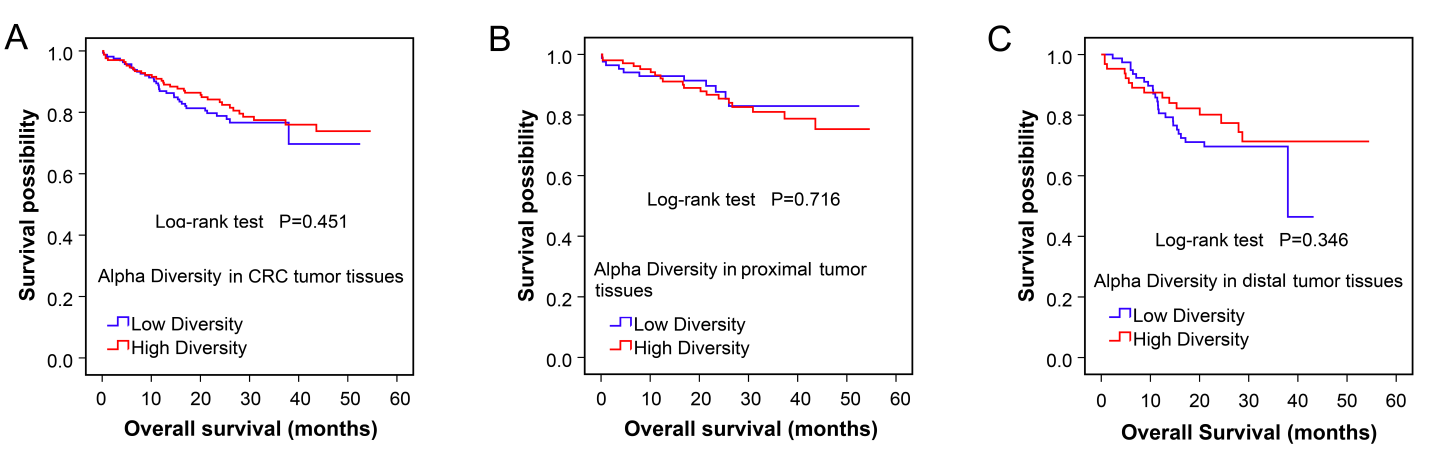


**Figure S1.** Effect of alpha diversity in tumor tissues on overall survival.

Kaplan-Meier curves for colorectal cancer-overall survival according to the relative abundance of alpha diversity in CRC tumor tissues (A), in proximal tumor tissues (B), distal tumor tissues (C). We define the level of alpha diversity (high, low) based on the average abundance in tumor tissues. CRC, colorectal cancer
